# Effect of Cu Content on Performance of Sn-Zn-Cu Lead-Free Solder Alloys Designed by Cluster-Plus-Glue-Atom Model

**DOI:** 10.3390/ma14092335

**Published:** 2021-04-30

**Authors:** Jialong Qiu, Yanzhi Peng, Peng Gao, Caiju Li

**Affiliations:** Faculty of Materials Science and Engineering, Kunming University of Science and Technology, Kunming 650093, China; qiujialong111@163.com (J.Q.); pengyanzhi1230@163.com (Y.P.)

**Keywords:** Sn-Zn solder alloy, cluster-plus-glue-atom-model, mechanical property, microstructure, interfacial reaction

## Abstract

The mechanical properties of solder alloys are a performance that cannot be ignored in the field of electronic packaging. In the present study, novel Sn-Zn solder alloys were designed by the cluster-plus-glue-atom (CPGA) model. The effect of copper (Cu) addition on the microstructure, tensile properties, wettability, interfacial characterization and melting behavior of the Sn-Zn-Cu solder alloys were investigated. The Sn_29_Zn_4.6_Cu_0.4_ solder alloy exhibited a fine microstructure, but the excessive substitution of the Cu atoms in the CPGA model resulted in extremely coarse intermetallic compound (IMC). The tensile tests revealed that with the increase in Cu content, the tensile strength of the solder alloy first increased and then slightly decreased, while its elongation increased slightly first and then decreased slightly. The tensile strength of the Sn_29_Zn_4.6_Cu_0.4_ solder alloy reached 95.3 MPa, which was 57% higher than the plain Sn-Zn solder alloy, which is attributed to the fine microstructure and second phase strengthening. The spreadability property analysis indicated that the wettability of the Sn-Zn-Cu solder alloys firstly increased and then decreased with the increase in Cu content. The spreading area of the Sn_29_Zn_0.6_Cu_0.4_ solder alloy was increased by 27.8% compared to that of the plain Sn-Zn solder due to Cu consuming excessive free state Zn. With the increase in Cu content, the thickness of the IMC layer decreased owing to Cu diminishing the diffusion force of Zn element to the interface.

## 1. Introduction

In the past few decades, conventional Sn-Pb solders have been extensively used in the electronic industry due to their excellent soldering properties and low cost. Pb is toxic and it is hazardous not only to the environment, but also to human health [1,2,3]. This toxicity has also become the main driving force to promote the development of lead-free solder alloys. Eutectic Sn-8.8Zn solder alloy has a relatively low melting point (198.5 °C), which is quite close to that of the traditional eutectic Sn-Pb solder alloy (183 °C). This means that when applying lead-free solder to the field of electronic packaging, the soldering process and apparatus developed for eutectic Sn-Pb solder alloy over the past few years can be used for references. In addition, the Sn-Zn eutectic alloy has been widely considered for other reasons, such as low cost, decent mechanical properties and sufficient supply [4,5]. However, the Sn-Zn solder alloy also has some undesirable characteristics, such as the existence of the coarse primary Zn phase in the microstructure. At present, adding trace alloying elements, such as Ag, Ni and RE, is a feasible method for refining the primary Zn phase [6,7,8].

Since the reliability of the solder joints mainly depends on the ability of the solder alloy to withstand deformation, the mechanical properties have received significant attention. A novel lead-free solder should possess excellent mechanical properties to meet the reliability requirements of the electronic industry [9,10,11]. Therefore, it is advisable to further improve the mechanical properties of the Sn-Zn solder. Pandey et al. [12] studied the effect of different Cu content on the mechanical properties of Sn-14 at.%Zn and showed that the yield strength of Sn-14.9 at.%Zn-0.841 at.% Cu reached the highest 60 MPa due to the presence of Cu-Zn IMC. In addition, many elements have also been incorporated into Sn-Zn solder alloy to improve the mechanical properties, such as Bi, Ag, Cr and rare earth (RE) [8,13,14,15].

The intermetallic compound (IMC) layer at the solder joint interface is intimately related to the solder joint reliability. Excessive IMC generation at the solder joint interface will be detrimental to the stability of the solder joint. In order to improve the interface characteristics, many researchers have added trace elements to suppress the interfacial reaction, such as Cr, Al and Ni [16,17,18].

Cu is widely used on printed circuit boards and is an important material in electronic devices. In addition, it is relatively cheap and easily available. Cu was also an element extensively used in lead-free solders. According to the Cu-Zn binary diagram, Cu and Zn can form Cu-Zn IMC in a wide composition range. Therefore, the addition of Cu is expected to refine the microstructure and improve the properties of Sn-Zn solder alloys. Some previous reports also indicate that Cu is conducive to improving the performance of Sn-Zn solder alloys [19,20].

Alloy materials with excellent performance are generally obtained by co-alloying multicomponent alloys. For the design of multicomponent alloys, it is often difficult to find a suitable method. Dong et al. proposed the cluster-plus-glue-atom (CPGA) theoretical model to describe a short-range order structure of amorphous alloys and solid solution alloy [21,22,23]. The CPGA model is mainly composed of two parts, one of which is the cluster part, and the other is the glue atom part. The cluster part is a nearest-neighbor coordination polyhedral, which describes short-range order structural features and generally consists of elements with relatively negative enthalpy of mixing. The glue atoms are located in polyhedral interstitial sites between clusters and are usually composed of elements with weak mixing enthalpy. Therefore, the CPGA model can be expressed by cluster formula [cluster] (glue atoms) [24,25]. This method can be used to understand the structure of binary and multicomponent alloys from a new perspective and provide effective theoretical guidance for the composition design of multicomponent alloys. At present, the model has been successfully applied to Cu-based alloys [26,27], Ni-based fillers [24] and Sn-based lead-free solders [25,28].

In this paper, novel Sn-Zn-Cu solder alloys were designed by the CPGA model. The effect of Cu addition on the microstructure, mechanical properties, melting behavior, wettability and interfacial characterization of the novel Sn-Zn-Cu solder alloys were investigated.

## 2. Materials and Methods

The crystal structures of Sn and Zn were analyzed based on the CPGA model. The cluster formula of [Sn-Sn_10_]Sn_5_ + [Sn-Sn_10_]Zn_5_Sn_2_ was obtained to describe the eutectic point Sn_85.1_Zn_14.9_ (at.%) [21]. Cu elements were added into Sn-Zn eutectic solder alloy to form Sn-Zn-Cu ternary solder alloys. According to the principle of strong interaction and mixing enthalpy theory, the enthalpy ΔH of Sn-Cu and Zn-Cu are 7 KJ/mol and 1 KJ/mol, respectively [29]. As the enthalpy of mixing between Cu and Zn is relatively low and the interaction is strong, part of the Zn atoms was replaced by Cu. According to the cluster formula above and the relationship between the elements, a series of ternary solder alloys were designed by replacing Zn with different amounts of Cu, as listed in Table 1, i.e., [Sn-Sn_10_]Sn_5_ + [Sn-Sn_10_]Zn_5−x_Cu_x_Sn_2_ (x = 0, 0.2, 0.4, 0.6, 0.8, 1.0).

The specific composition Sn-Zn-Cu solder alloys in Table 1 were prepared by melting pure Sn (99.99 wt.%), Zn (99.99 wt.%), Cu (99.99 wt.%). Prior to smelting, the elemental mixtures were accurately weighed and sealed in quartz tubes with a vacuum of 10^−4^ Pa. The quartz tube was heated to 800 °C in a muffle furnace (Siomm SXL-1400, Shanghai, China) and held for 3 h. During the heating process, the tubes were periodically mechanically shaken to obtain the compositional homogenization of elemental mixtures. After heating, the quartz tubes were water-quenched at about 300 °C.

For microstructure analysis of solder alloys, the samples were grinded with silicon-carbide papers. The polished samples were ultrasonically cleaned with deionized water and ethanol. Finally, samples were etched with corrosion solution (4 vol% HNO_3_ + 96 vol% CH_3_CH_2_OH solution) for about 5 s. After metallographic treatment, microstructures of specimens were observed by scanning electron microscope (SEM, Tescan VEGA 3, Brno, Czech Republic).

The thermal behavior of as-cast alloy was measured by differential scanning calorimetry (DSC, TA Instruments DSC 25, New Castle, DE, USA) by using 10 mg which was obtained from the as-cast alloy. Alloy samples were sealed in a hermetic aluminum pan and were heated in nitrogen atmosphere from the ambient temperature to 250 °C and held for 5 min at 250 °C then cooled to room temperature. The heating and cooling rates were both 10 °C/min during the experiment.

Wetting tests were performed according to Chinese National Standard GB/T 11364-2008. The oxygen-free pure copper substrates for spreadability property test with size of 40 × 40 × 1 mm^3^ were prepared. Copper substrates were fully polished with a series of SiC papers from 600 to 5000 grit first. Then, copper substrates were immersed in 10 vol% HCl for 20 s to remove the oxide film, followed by ultrasonically cleaning with ethanol. The each of solder alloys with weight of 0.3 ± 0.005 g were cut from cylindrical master alloys. Then, the solder balls were prepared by soaking the solder alloys in a stainless-steel spoon which filled with heated liquid rosin. The spoon was cooled quickly in water to make the solder ball when the solder melts to form the ball. The solder balls were placed on Cu substrate which was covered with rosin flux and then heated at 250 °C for 2 min in the reflow oven (Tinyo TYR108N-C, Beijing, China). The spreading area was measured by Auto CAD software (Auto CAD 2018, 2018, Autodesk, San Rafael, CA, USA). The morphology of the interface between solder and copper substrates was observed with SEM.

The specimens for the tensile test were mechanically ground with SiC papers from 800 to 2000 grain size to polish the cut marks on the sample surface to ensure a more precise measurement before the tensile tests. Subsequently, tensile tests were conducted by a universal testing machine (Shimadzu AG-X-100kN, Kyoto, Japan) with a constant deformation rate of 1 mm/min. At least three specimens were used for the tensile strength test of Sn-Zn-Cu solder alloys. The fracture surfaces of the specimens were observed by SEM.

## 3. Results and Discussion

### 3.1. Microstructure of Solder Alloys

**Figure 1:**
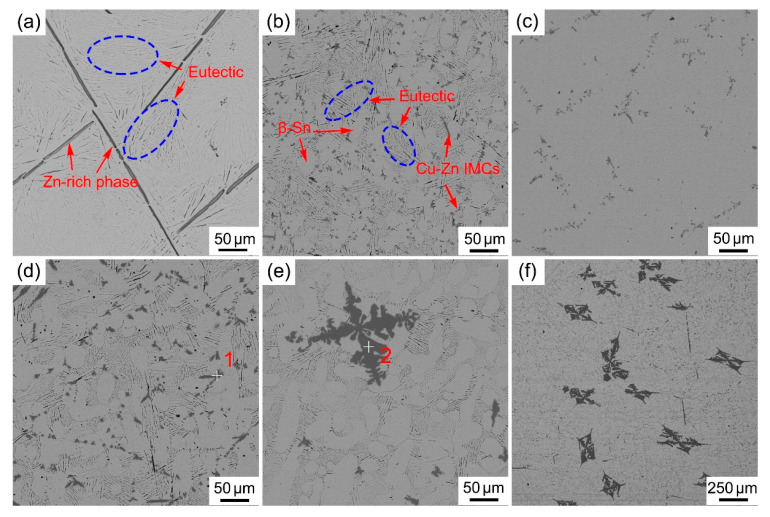
SEM microstructure of the Sn-Zn-Cu solder alloys and corresponding EDS results: (**a**–**f**) (Sn_29_Zn_5-x_Cu_x_, x = 0, 0.2, 0.4, 0.6, 0.8, 1.0).

The typical microstructure of as-cast Sn_29_Zn_5-x_Cu_x_ (x = 0, 0.2, 0.4, 0.6, 0.8, 1.0) solder alloys are shown in Figure 1. The plain Sn_29_Zn_5_ solder alloy in Figure 1a included dark primary Zn phases, eutectic α-Zn and gray β-Sn matrix. Due to local variation of the solidification conditions, rodlike primary Zn phases are randomly distributed in β-Sn matrix, and acicular α-Zn phases are uniformly distributed in β-Sn matrix, which exhibits the eutectic structure.

**Figure 2:**
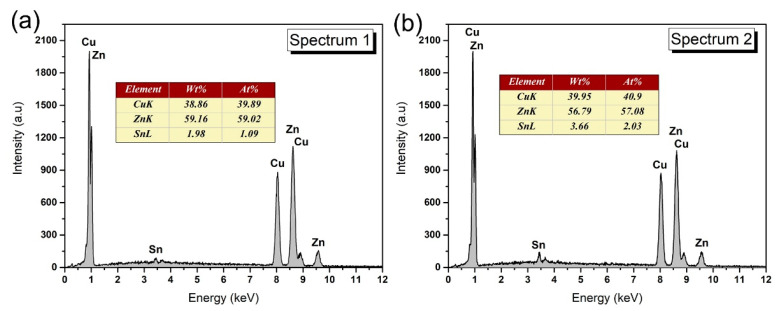
Composition results of point 1 and 2 in Figure 1d,e. (**a**) spectrum 1; (**b**) spectrum 2.

With the addition of the third element Cu, as shown in Figure 1b–f, the new dark gray phases appeared in the matrix and were accompanied by the β-Sn phases with irregular cellular morphology. In addition, the primary Zn phase also disappeared. According to the binary phase diagrams of the Sn-Cu and Zn-Cu, Cu could react with Sn and Zn to form IMC; because of the greater reactivity of Zn, Cu will react with Zn in preference to Sn [30]. Therefore, the newly emerging corresponding composition is Cu-Zn IMC. This speculation was also proved by the EDS in Figure 2. According to the composition results, it can be inferred that the intermetallic compound was Cu_5_Zn_8_.

It is not difficult to find from Figure 1b,c that the addition of a minute amount of Cu makes the coarse Zn-rich phase disappear, and then a more uniformly distributed eutectic structure appears. Therefore, a small amount of Cu can refine the structure. The design based on the CPGA model was a process in which Cu atoms gradually replace Zn atoms, and the new Cu_5_Zn_8_ phase formed further consumes Zn atoms, resulting in the appearance of dendritic β-Sn phase. With the increase in Cu, the eutectic phase gradually decreased. In Figure 1e,f, a coarse dendritic Cu_5_Zn_8_ phase appeared, the size of which exceeded 100 μm in the length direction. The microstructure with low-magnification was shown in Figure 1f. Many large Cu_5_Zn_8_ grains have been observed. The appearance of a large size of this phase will deteriorate its properties. It can be seen that the microstructure was coarsened by the addition of excessive Cu.

### 3.2. Mechanical Properties of Solder Alloys

**Figure 3:**
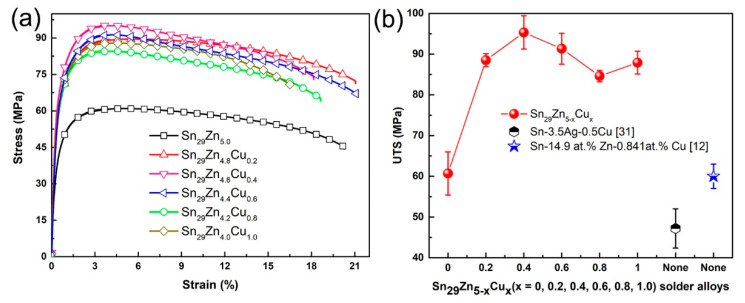
Comparison of the tensile properties of the Sn_29_Zn_5-x_Cu_x_ (x = 0, 0.2, 0.4, 0.6, 0.8, 1.0) solder alloys: (**a**) stress–strain curves, (**b**) UTS.

The stress–strain curves of the Sn_29_Zn_5-x_Cu_x_ (x = 0, 0.2, 0.4, 0.6, 0.8, 1.0) solder alloys are shown in Figure 3a. The variation tendency of the ultimate tensile strength (UTS) of solder alloys were summarized in Figure 3b. The UTS of the Sn_29_Zn_5_ solder alloy was approximately 60.7 MPa. The UTS of the Sn-Zn-Cu solder alloys was obviously increased, especially the tensile strength of the Sn_29_Zn_4.6_Cu_0.4_ solder alloy reached the highest 95.3 MPa, which was 57% higher than of the plain Sn_29_Zn_5_ solder alloy, 101% higher than the Sn-3.5Ag-0.5Cu solder alloy (47.2 MPa) that is used more often in practice and 58.8% higher than the Sn-14.9 at.%Zn-0.841 at.% Cu (60 MPa) reported by Pandey et al. [12,31]. For the elongation, with the increase in Cu content, the elongation first increased slightly and then decreased. When a small amount of Cu was added, the elongation almost remained unchanged or slightly increased, while the elongation of the Sn_29_Zn_4.0_Cu_1.0_ solder alloy was 16.6% and decreased by 3.7%. It can be seen that the addition of Cu has little effect on the elongation.

As mentioned above, Cu and Zn will form intermetallic compounds when Cu is added, which can be distributed in the matrix as second phase particles to hinder dislocation movement when plastic deformation occurs and form the effect of dispersion strengthening. In addition, in the solder alloy with Cu added, the originally thick Zn-rich phase was also broken and disappeared, and then the eutectic Zn phase with fine needle shape and uniform distribution appeared, which could also play a similar effect of dispersion strengthening on the matrix [3]. The improvement in strength by dispersion strengthening can be approximated as [32,33].

σ = Gb/λ(1)

where σ is the yield stress (MPa), G is the shear modulus of the material matrix (Pa), b is Burgers vector (nm) and λ refers to the distance between the dispersed particles (μm). When a small amount of Cu was added, a relatively small Cu_5_Zn_8_ particles were formed in the matrix and the coarse primary Zn phases were refined, showing a low λ value, thus improving the strength of solder alloy. However, with the increase in Cu content, coarse Cu_5_Zn_8_ particles appeared in the microstructure, as shown in Figure 1e,f. The edge of coarse second phase particles may form some weak bonding interface with the matrix, thus becoming the site for crack initiation and propagation, thus reducing its strength. A similar phenomenon was reported by Lu et al. [2]. They added Zr to Sn-3.0Ag-0.5Cu solder alloy and found that Zr refined Ag_3_Sn particles so that Ag_3_Sn particles have smaller size and shorter spacing, thus increasing the effect of dispersion strengthening and improving the mechanical properties of the original solder alloy.

As for the elongation of the material, with the increase in Cu content, the elongation first increases and then decreases, which can also be explained from the perspective of microstructure. When a small amount of Cu was added, the thick and relatively brittle Zn-rich phase disappears, and a more detailed acicular eutectic phase appears. The thick and Zn-rich phase is more likely to become a site for crack initiation and expansion, which is unfavorable to the plasticity of solder alloy, so the disappearance of this coarse phase makes the plasticity slightly improved. However, with the increase in Cu content, the bulky Cu_5_Zn_8_ particles appeared again, which significantly increased the brittleness of the solder and thus made the plasticity of the solder alloy appear to decrease again.

**Figure 4:**
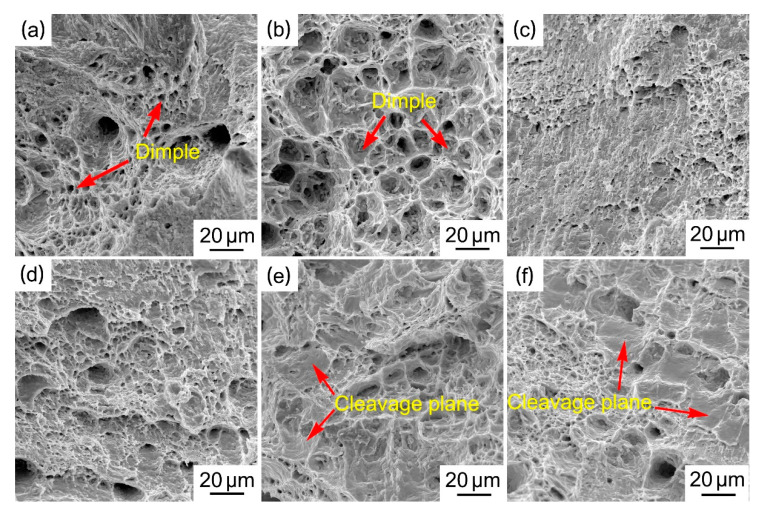
SEM micrographs of tensile fracture of the Sn-Zn-Cu solder alloys: (**a**–**f**) Sn_29_Zn_5-x_Cu_x_ (x = 0, 0.2, 0.4, 0.6, 0.8, 1.0) solder alloys.

Figure 4 shows the fracture surface of the Sn_29_Zn_5-x_Cu_x_ (x = 0, 0.2, 0.4, 0.6, 0.8, 1.0) solder alloys after tensile test.

As can be seen from Figure 4a, the fracture of the Sn_29_Zn_5_ solder alloy has obvious deep and larger dimples, indicating that it is a typical ductile fracture. It is well known that the plasticity of the material increases with the increase in the size and relative area fraction of the dimple, and the brittleness of the material enhances with the increase in the size and area of the cleavage under the same fracture condition [34]. The fracture of the Sn_29_Zn_4.8_Cu_0.2_ solder alloy had more dimples than Sn_29_Zn_5_, which has excellent elongation. The fracture morphology of the Sn_29_Zn_4.2_Cu_0.8_ solder alloy shows a cleavage plane. With the increase in Cu content, the relative area fraction of cleavage plane exceeds that of dimple, which indicates that ductile fracture has been partially replaced by brittle fracture. Figure 1e,f shows the appearance of coarse Cu_5_Zn_8_ intermetallic compounds in the microstructure. The Cu_5_Zn_8_ phase is a hard and brittle phase. During the plastic deformation process, the slip surface of the Cu_5_Zn_8_ phase cannot slide freely in the proper direction, which shows low plastic deformation ability. On the other hand, the coarse Cu_5_Zn_8_ IMCs hinder the dislocation movement, leading to local stress concentration, which increases the brittleness of the solder alloy and thus reduces the plasticity of the solder. It is not difficult to observe from Figure 4 that the fracture mechanism of the Sn_29_Zn_5-x_Cu_x_ (x = 0, 0.2, 0.4, 0.6, 0.8, 1.0) solder alloys changed from a ductile fracture to a mixed fracture mode in which ductile and brittle fracture co-existed, which was consistent with the change trend of elongation.

### 3.3. Wettability of Solder Alloys

**Figure 5:**
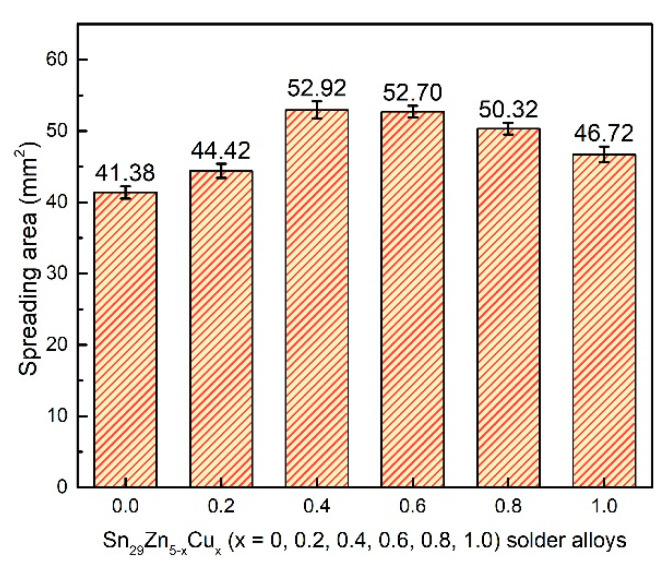
Spreading area of the Sn_29_Zn_5-x_Cu_x_ (x = 0, 0.2, 0.4, 0.6, 0.8, 1.0) solder alloys.

The wettability of the solder alloy refers to the ability of the molten liquid alloy solder to spread on the surface of the solid substrate. Whether the molten solder can form good wetting with the substrate is the key to completing the soldering. Figure 5 shows the variations of spreading area of the Sn_29_Zn_5-x_Cu_x_ (x = 0, 0.2, 0.4, 0.6, 0.8, 1.0) solder alloys as a function of the Cu contents.

From Figure 5, it can be noticed that as the Cu content increases, the spreading area appears to increase first and then decrease again. Among them, the spreading area of the Sn_29_Zn_4.6_Cu_0.4_ solder alloy reached the highest 52.92 mm^2^, which was 27.8% higher than the original Sn_29_Zn_5_. The Cu addition can improve the spreading area, but larger Cu additions negate the wettability. Previous reports indicated that the poor wettability of the Sn-Zn solder alloy is due to the high activity of Zn, which leads to quite a large surface tension of the molten solder. The Zn in the solder is also easily oxidized during the soldering process, and the generated ZnO coats the surface of the solder and hinders the fluidity of the molten solder [15]. After adding Cu, the Zn in Sn-Zn solder can be refined and formed Cu-Zn IMC, which consumes excessive free state Zn. Then, it can reduce the generation of ZnO oxide, and alleviate surface tension of liquid solder. In addition, the addition of excessive Cu slightly reduces the wetting ability of the solder. One possible explanation is that the large blocks Cu-Zn IMC precipitated in the solder (Figure 1f) have a negative effect on the flow of the molten solder. Therefore, the benefit term of wettability owing to the incorporation of Cu is shaded and the ability to improve wettability is weakened in this case.

### 3.4. Interfacial Characterization of Solder Joint

**Figure 6:**
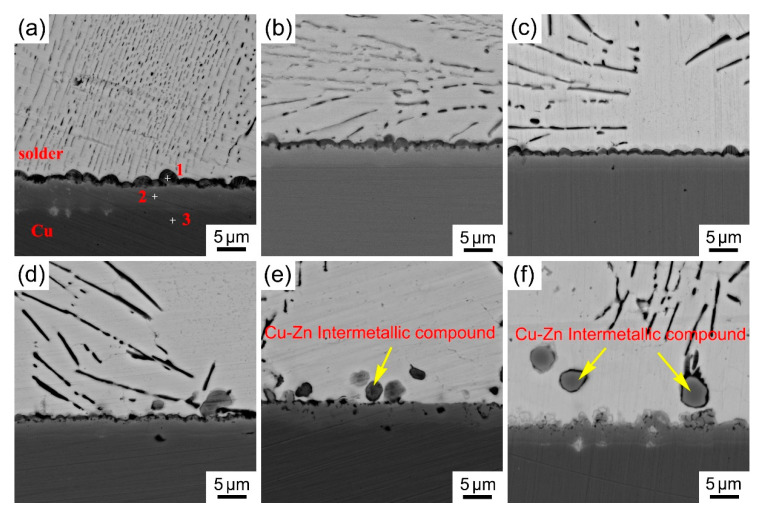
Interfacial morphology of the Sn-Zn-Cu solder joints after soldering (**a**–**f**) (Sn_29_Zn_5-x_Cu_x_, x = 0, 0.2, 0.4, 0.6, 0.8, 1.0).

As all know, the reliability of solder joints is also a major concern in electronics assemblies. The morphology and thickness of the interfacial reaction layer directly affect the mechanical properties of the solder joints. Figure 6 shows typical interface morphology of the Sn_29_Zn_5-x_Cu_x_ (x = 0, 0.2, 0.4, 0.6, 0.8, 1.0) solder alloys after soldering.

Two reaction layers were observed. The thick reaction layer adjacent to the Cu substrates is planar, and the other thinner layer next to the solder is scallop type. The compositions of the different points in Figure 6a are summarized in Table 2. The composition of the planar reaction layer (point 2) is Cu:Zn:Sn = 35.4:63.6:0.9 (at.%) and the scallop type reaction layer (point 1) is Cu:Zn:Sn = 18.7:71.2:10.1 (at.%). In terms of the compositions’ results, and the Sn-Zn-Cu ternary phase diagram [35], the planar reaction layer was identified as Cu_5_Zn_8_. Regarding the element composition of the scallop type reaction layer, it seems that the reaction layer is Cu-(Zn, Sn)_5_ intermetallic based on the Cu:(Zn + Sn) atomic ratio of 18.7:(71.2 + 10.1). That is, Sn may dissolve in the CuZn_5_ compound and partially substitute the Zn atom site to form Cu-(Zn, Sn)_5_. Some previous studies have reported similar morphology of reaction layer [18,36,37,38]. In addition, when the Cu contents reached up to x = 0.8, it could be found that the scallop type Cu-(Zn, Sn)_5_ IMC layer disappeared and some other Cu-Zn intermetallic compounds were also observed near the edge of the interfacial reaction layer. One possible reason for the absence of the Cu-(Zn, Sn)_5_ IMC layer was that there were not enough Zn atoms to react with Cu to form Cu-(Zn, Sn)_5_ since the depletion of Zn atoms in the bulk solder.

**Figure 7:**
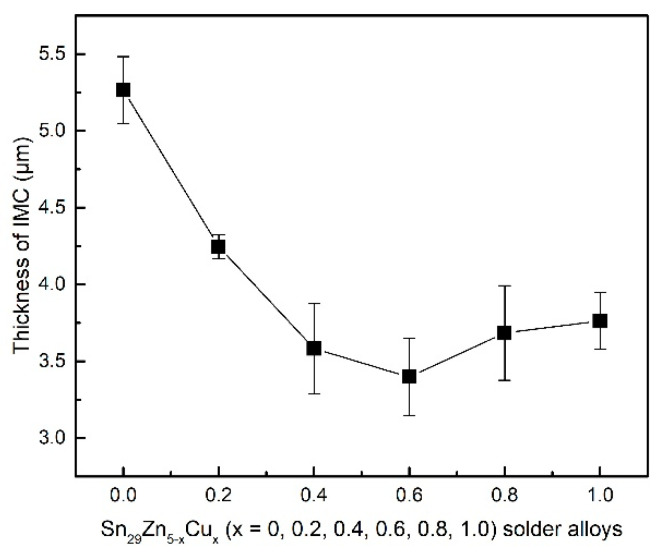
The average thickness of the IMC layers formed at Sn_29_Zn_5-x_Cu_x_/Cu interface after soldering.

The average thickness of the IMC layer was obtained by dividing the area of the IMC layer by its length. The results are shown in Figure 7.

With the increase in Cu content, the thickness of the IMC layer decreases. For the Sn_29_Zn_0.4_Cu_0.6_/Cu interface, the thickness of the IMC layer was only 3.40 μm, which was 35% lower than that of the Sn_29_Zn_5_/Cu (5.27 μm). This result reveals that Cu can inhibit the formation and growth of the IMC layer. As demonstrated above, Cu-Zn IMC were formed in the bulk solder after adding Cu due to the strong interaction between Cu and Zn. Some Cu-Zn intermetallic compounds have also been observed at the boundary of the interfacial reaction layer. Previous reports [18,37] indicated that the growth of interfacial reaction layer is mainly determined by the diffusion of the Cu and Zn atoms and the diffusion of Zn atoms to Cu substrate is the major contributor. The Cu in bulk solder reduces the activity of Zn and suppresses the diffusion driving force of Zn toward the interface, thus inhibiting the growth interfacial reaction layer. Liu et al. [37] added Cu to the Sn-8Zn-3Bi solder alloy and found that with the increase in Cu content, the activation energy of the IMC growth gradually increased, which effectively inhibited the growth of the interfacial IMC. Liu et al. [18] found that adding Ni to the Sn-8Zn-3Bi solder alloy reduces the thickness of the interface IMC layer due to Ni-Zn IMC formed in the bulk solder. As the interfacial layer is brittle and the thermal expansion coefficient of the substrate and the interfacial layer is different, the excessive interfacial reaction layer will deteriorate solder joint reliability [39].

### 3.5. Thermal Behavior of Solder Alloys

**Figure 8:**
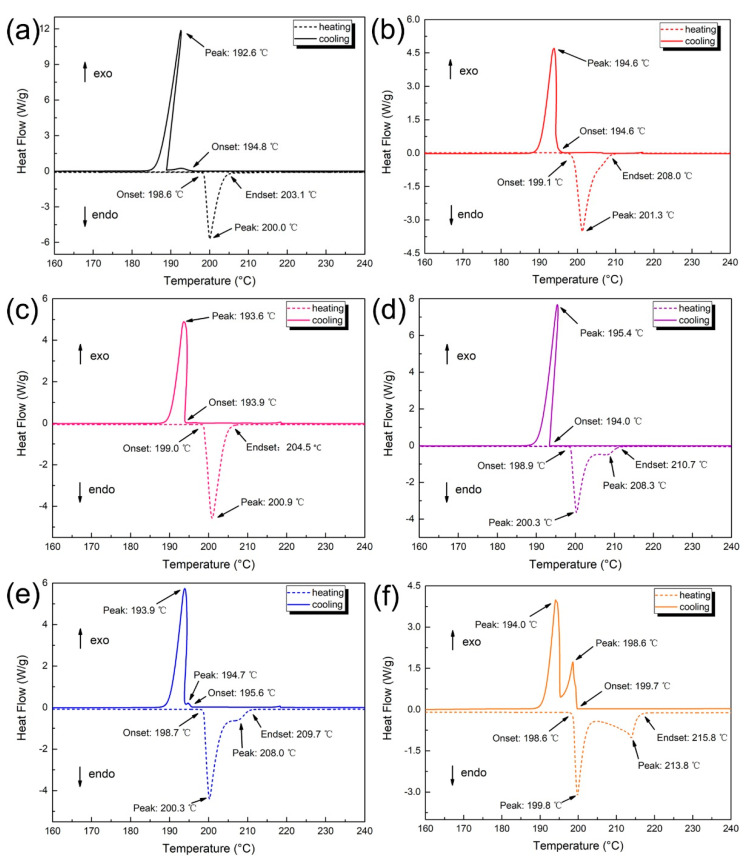
DSC curves of the Sn-Zn-Cu solder alloys (**a**–**f**) (Sn_29_Zn_5-x_Cu_x_, x = 0, 0.2, 0.4, 0.6, 0.8, 1.0).

In order to adapt to the mature soldering process and equipment that has been developed for a long time, the melting characteristics of the solder have to be considered. The melting point of lead-free solder should be as close as possible to the melting point of the Sn-Pb solder (183 °C). The DSC curves of heating and cooling of solder alloys and the measured data are shown in Figure 8.

It can be seen from Figure 8 that the only one endothermic peak of the Sn_29_Zn_5-x_Cu_x_ (x = 0, 0.2, 0.4) solder alloys was observed (T_peak_), while two endothermic peaks were observed for Sn_29_Zn_5-x_Cu_x_ (x = 0.6, 0.8, 1.0) solder alloys, which correspond to eutectic and β-Sn phase in the microstructure. The pasty range, undercooling and peak temperature of these solder alloys were calculated. According to the results, with the increase in Cu content, the T_peak_ value is only slightly changed, which is about 1 °C, indicating that the Cu content has little reliance on T_peak_ value. The starting point of the peak is the temperature at which the phase transition begins, represented as T_onset_, and the end temperature is represented as T_endset_. During heating, T_onset_ is called the solidus temperature of the solder alloys and T_endset_ is called the liquidus point [36]. According to Figure 8, the Cu content has little effect on the temperature and its change does not exceed 2.0 °C, but it has a greater effect on the liquidus temperature, with the highest change reaching 7.6 °C.

The difference between T_onset_ and T_endset_ during the endothermic process is usually considered the melting range of the solder alloy. The melting range of the Sn_29_Zn_4.6_Cu_0.4_ solder alloy is increased from 4.5 °C to 5.5 °C, but it is still lower than the melting range of Sn-Pb (11.5 °C). However, the melting range of the Sn_29_Zn_4.0_Cu_1.0_ solder alloy increased by 7.5 °C to 12.0 °C. The addition of too much Cu increases the melting range of the solder alloy, which may be caused by the following two reasons. On the one hand, the Zn content of the component designed by the CPGA model is a process of being gradually replaced by Cu, which gradually deviates from the eutectic point and becomes a hypoeutectic point, thus increasing the melting range. On the other hand, it has been mentioned that Cu is superior to Sn and easy to combine with Zn to form Cu-Zn IMC [30], which further reduces the content of Zn in the melt, thus reducing the formation of eutectic structure, and then the β-Sn phase appears. This corresponds to multiple endothermic peaks and exothermic peaks in the DSC curve. Therefore, the increase in the melting range can be attributed to the decrease in the Zn before the solidification of the Sn-Zn system alloy. However, a small amount of Cu will not greatly increase the melting range of the solder alloy, and it has certain advantages compared to the melting range of the Sn-Pb (11.5 °C) solder alloy [7].

## 4. Conclusions

In the present study, the microstructure, tensile properties, wettability, interfacial characterization and melting behavior of the Sn_29_Zn_5-x_Cu_x_ (x = 0, 0.2, 0.4, 0.6, 0.8, 1.0) solder alloys designed by CPGA model were investigated. The conclusions are drawn as follows:

(1) The microstructure of the Sn-Zn-Cu solder alloy consists of the α-Zn, β-Sn and Cu_5_Zn_8_ phases. The microstructure of the Sn-Zn solder alloy was refined with the addition of Cu, but excessive addition of Cu will result in the appearance of larger size intermetallic compounds.
(2) The tensile test results show that the UTS value increases with the increase in Cu content and the UTS value of the new Sn_29_Zn_4.6_Cu_0.4_ solder alloy is increased by 57% to 95.3 MPa compared with the original Sn-Zn solder. The improved mechanical properties can be attributed to the fine microstructure and dispersion strengthening of the second phase.
(3) The novel Sn_29_Zn_0.6_Cu_0.4_ solder obtains the largest spreading area among the Sn_29_Zn_5-x_Cu_x_ (x = 0, 0.2, 0.4, 0.6, 0.8, 1.0) solder alloys, which was 27.8% higher than that of the plain Sn-Zn solder alloy. However, with the continuous addition of Cu, the spread area tends to decrease. With the increase in the Cu content, the thickness of the IMC layer decreased owing to Cu slowing the diffusion force of Zn to the interface.

## Figures and Tables

**Table 1:** Composition of the Sn-Zn-Cu solder alloys designed by CPGA model.

No.	Cluster Formula	Alloy Composition	
		at.%	wt.%
(a)	[Sn-Sn_10_]Sn_5_ + [Sn-Sn_10_]Zn_5_Sn_2_	Sn_85.3_Zn_14.7_	Sn_91.33_Zn_8.67_
(b)	[Sn-Sn_10_]Sn_5_ + [Sn-Sn_10_]Zn_4.8_Cu_0.2_Sn_2_	Sn_85.29_Zn_14.12_Cu_0.59_	Sn_91.33_Zn_8.33_Cu_0.34_
(c)	[Sn-Sn_10_]Sn_5_ + [Sn-Sn_10_]Zn_4.6_Cu_0.4_Sn_2_	Sn_85.29_Zn_13.53_Cu_1.18_	Sn_91.34_Zn_7.98_Cu_0.68_
(d)	[Sn-Sn_10_]Sn_5_ + [Sn-Sn_10_]Zn_4.4_Cu_0.6_Sn_2_	Sn_85.30_Zn_12.94_Cu_1.76_	Sn_91.37_Zn_7.63_Cu_1.00_
(e)	[Sn-Sn_10_]Sn_5_ + [Sn-Sn_10_]Zn_4.2_Cu_0.8_Sn_2_	Sn_85.3_Zn_12.35_Cu_2.35_	Sn_91.36_Zn_7.29_Cu_1.35_
(f)	[Sn-Sn_10_]Sn_5_ + [Sn-Sn_10_]Zn_4.0_Cu_1.0_Sn_2_	Sn_85.29_Zn_11.76_Cu_2.95_	Sn_91.37_Zn_6.94_Cu_1.69_

**Table 2:** Composition results of EDS at different location in Figure 4a.

Point	Sn (at.%)	Zn (at.%)	Cu (at.%)
1	10.1	70.2	18.7
2	0.9	63.6	35.4
3	-	-	100

## Data Availability

Data are available on request.

## References

[1] Jaffery H.A., Sabri M.F.M., Said S.M., Hasan S.W., Sajid I.H., Nordin N.I.M., Megat Hasnan M.M.I., Shnawah D.A., Moorthy C.V. (2019). Electrochemical corrosion behavior of Sn-0.7Cu solder alloy with the addition of bismuth and iron. J. Alloys Compd..

[2] Lu T., Yi D., Wang H., Tu X., Wang B. (2019). Microstructure, mechanical properties, and interfacial reaction with Cu substrate of Zr-modified SAC305 solder alloy. J. Alloys Compd..

[3] Ren G., Collins M.N. (2017). The effects of antimony additions on microstructures, thermal and mechanical properties of Sn-8Zn-3Bi alloys. Mater. Des..

[4] Liu J.C., Park S., Nagao S., Nogi M., Koga H., Ma J.S., Zhang G., Suganuma K. (2015). The role of Zn precipitates and Cl^−^ anions in pitting corrosion of Sn-Zn solder alloys. Corros. Sci..

[5] Huang N., Hu A., Li M., Mao D. (2013). Influence of Cr alloying on the oxidation resistance of Sn–8Zn–3Bi solders. J. Mater. Sci. Mater. Electron..

[6] El-Daly A.A., Hammad A.E. (2010). Effects of small addition of Ag and/or Cu on the microstructure and properties of Sn–9Zn lead-free solders. Mater. Sci. Eng. A.

[7] El-Daly A.A., Hammad A.E., Al-Ganainy G.S., Ibrahiem A.A. (2014). Design of lead-free candidate alloys for low-temperature soldering applications based on the hypoeutectic Sn–6.5Zn alloy. Mater. Des..

[8] Chen W., Xue S., Wang H., Hu Y., Wang J. (2010). Effects of rare earth Ce on properties of Sn-9Zn lead-free solder. J. Mater. Sci. Mater. Electron..

[9] Wei C., Liu Y.C., Yu L.M., Chen H., Wang X. (2010). Effects of Al on the failure mechanism of the Sn-Ag-Zn eutectic solder. Microelectron. Reliab..

[10] Luo T., Hu A., Hu J., Li M., Mao D. (2012). Microstructure and mechanical properties of Sn-Zn-Bi-Cr lead-free solder. Microelectron. Reliab..

[11] Mohd Nasir S.S., Yahaya M.Z., Erer A.M., Illés B., Mohamad A.A. (2019). Effect of TiO2 nanoparticles on the horizontal hardness properties of Sn-3.0Ag-0.5Cu-1.0TiO_2_ composite solder. Ceram. Int..

[12] Pandey P., Tiwary C.S., Chattopadhyay K. (2019). Effects of Cu and In Trace Elements on Microstructure and Thermal and Mechanical Properties of Sn-Zn Eutectic Alloy. J. Electron. Mater..

[13] El-Daly A.A., Hashem H.A., Radwan N., El-Tantawy F., Dalloul T.R., Mansour N.A., Abd-Elmoniem H.M., Lotfy E.H. (2016). Robust effects of Bi doping on microstructure development and mechanical properties of hypoeutectic Sn-6.5Zn solder alloy. J. Mater. Sci. Mater. Electron..

[14] Chen W.X., Xue S.B., Wang H., Wang J.X., Han Z.J., Gao L.L. (2010). Effects of Ag on microstructures, wettabilities of Sn-9Zn-xAg solders as well as mechanical properties of soldered joints. J. Mater. Sci. Mater. Electron..

[15] Chen X., Hu A., Li M., Mao D. (2008). Study on the properties of Sn–9Zn–xCr lead-free solder. J. Alloys Compd..

[16] Chen X., Hu A., Li M., Mao D. (2009). Effect of a trace of Cr on intermetallic compound layer for tin–zinc lead-free solder joint during aging. J. Alloys Compd..

[17] Lai R.S., Lin K.L., Salam B. (2009). Suppressing Growth of the Cu5Zn8 Intermetallic Layer in Sn-Zn-Ag-Al-Ga/Cu Solder Joints. J. Electron. Mater..

[18] Liu L.U., Zhou W., Li B.L., Wu P. (2010). Interfacial reactions between Sn-8Zn-3Bi-xNi lead-free solders and Cu substrate during isothermal aging. Mater. Chem. Phys..

[19] Das S.K., Sharif A., Chan Y.C., Wong N.B., Yung W.K.C. (2009). Influence of small amount of Al and Cu on the microstructure, microhardness and tensile properties of Sn-9Zn binary eutectic solder alloy. J. Alloys Compd..

[20] El-Daly A.A., Desoky W.M., Saad A.F., Mansor N.A., Lotfy E.H., Abd-Elmoniem H.M., Hashem H. (2015). The effect of undercooling on the microstructure and tensile properties of hypoeutectic Sn-6.5Zn-xCu Pb-free solders. Mater. Des..

[21] Dong C., Dong D.D., Wang Q. (2018). Chemical Units in Solid Solutions and Alloy Composition Design. Acta Metall. Sin..

[22] Ma Y.P., Dong D.D., Dong C., Luo L.J., Wang Q., Qiang J.B., Wang Y.M. (2015). Composition formulas of binary eutectics. Sci. Rep..

[23] Dong C., Wang Q., Qiang J.B., Wang Y.M., Jiang N., Han G., Li Y.H., Wu J., Xia J.H. (2007). From clusters to phase diagrams: Composition rules of quasicrystals and bulk metallic glasses. J. Phys. D Appl. Phys..

[24] Dong H., Xia Y., Xu X., Naz G.J., Hao X., Li P., Zhou J., Dong C. (2020). Performance of GH4169 brazed joint using a new designed nickel-based filler metal via cluster-plus-glue-atom model. J. Mater. Sci. Technol..

[25] Huang M.L., Yang Y.C., Chen Y., Dong C. (2016). Microstructure and mechanical properties of Sn-rich Au-Sn solders designed using cluster-plus-glue-atom model. Mater. Sci. Eng. A.

[26] Yu Q.X., Li X.N., Wei K.R., Li Z.M., Zheng Y.H., Li N.J., Cheng X.T., Wang C.Y., Wang Q., Dong C. (2019). Cu-Ni-Sn-Si alloys designed by cluster-plus-glue-atom model. Mater. Des..

[27] Zhang J., Wang Q., Wang Y., Wen L., Dong C. (2010). Highly corrosion-resistant Cu70(Ni, Fe, Mn, Cr)30 cupronickel designed using a cluster model for stable solid solutions. J. Alloys Compd..

[28] Huang M.L., Huang F.F., Yang Y.C. (2017). Composition design of Sn-rich Sn-Au-Ag solders using cluster-plus-glue-atom model. J. Mater. Sci. Mater. Electron..

[29] Takeuchi A., Inoue A. (2005). Classification of Bulk Metallic Glasses by Atomic Size Difference, Heat of Mixing and Period of Constituent Elements and Its Application to Characterization of the Main Alloying Element. Mater. Trans..

[30] Lee J.E., Kim K.S., Inoue M., Jiang J.X., Suganuma K. (2008). Effects of Ag and Cu addition on microstructural properties and oxidation resistance of Sn-Zn eutectic alloy. J. Alloys Compd..

[31] Chuang C.L., Tsao L.C., Lin H.K., Feng L.P. (2012). Effects of small amount of active Ti element additions on microstructure and property of Sn3.5Ag0.5Cu solder. Mater. Sci. Eng. A.

[32] Yang T.Q., Zhao X.C., Xiong Z.S., Tan W., Wei Y.H., Tan C.W., Yu X.D., Wang Y.C. (2020). Improvement of microstructure and tensile properties of Sn-Bi-Ag alloy by heterogeneous nucleation of β-Sn on Ag_3_Sn. Mater. Sci. Eng. A.

[33] Wang S.L., Murr L.E. (1980). Effect of prestrain and stacking-fault energy on the application of the Hall-Petch relation in fcc metals and alloys. Metallography.

[34] Zeng X.W., Liu Y.C., Zhang J.K., Liu Y., Hu X.W., Jiang X.X. (2020). Effect of rare earth Ce on the thermal behavior, microstructure and mechanical properties of Zn-30Sn-2Cu high temperature lead-free solder alloy. J. Mater. Sci. Mater. Electron..

[35] Chou C.Y., Chen S.W. (2006). Phase equilibria of the Sn-Zn-Cu ternary system. Acta Mater..

[36] Liu J.C., Zhang G., Wang Z.H., Ma J.S., Suganuma K. (2015). Thermal property, wettability and interfacial characterization of novel Sn-Zn-Bi-In alloys as low-temperature lead-free solders. Mater. Des..

[37] Liu L., Wu P., Zhou W. (2014). Effects of Cu on the interfacial reactions between Sn-8Zn-3Bi-xCu solders and Cu substrate. Microelectron. Reliab..

[38] Chou C.Y., Chen S.W., Chang Y.S. (2006). Interfacial reactions in the Sn-9Zn-(xCu)/Cu and Sn-9Zn-(xCu)/Ni couples. J. Mater. Res..

[39] Chen J., Shen J., Min D., Peng C. (2009). Influence of minor Bi additions on the interfacial morphology between Sn–Zn–xBi solders and a Cu layer. J. Mater. Sci. Mater. Electron..

